# DYSTHANASIA AND/OR FUTILE CARE IN THE INTENSIVE CARE UNITS OF A SPECIALTY HOSPITAL IN EL BAJío region, in Mexico

**DOI:** 10.1186/2197-425X-3-S1-A655

**Published:** 2015-10-01

**Authors:** EM Olivares-Durán, AM Madrigal-Arcos

**Affiliations:** Adult Intensive Care Unit, High Specialty Regional Hospital of El Bajío (HRAEB), León, Mexico; Department of Nursing and Obstetrics, University of Guanajuato, Campus León, León, Mexico

## Introduction

End-of-life care is emerging as a comprehensive area of expertise in the ICU and demands the same high level of knowledge and competence as all other areas of ICU practice¹.

## Objectives

To estimate the frequency with which dysthanasia and/or futile care is practiced in adult and pediatric intensive care units (ICUs) of a specialty hospital in El Bajío Region, in Mexico, and to identify the main factors associated with their occurrence.

## Methods

A survey on the “Factors Involved in Dysthanasia and/or Futile Care”, designed by the authors, was applied to medical and nursing staff of every ICUs of the High Specialty Regional Hospital of El Bajío (HRAEB) along a period of 5 months. Staff participation was voluntary and the forms were self-reported. The interviews were conducted individually, in a private setting, and within a context of confidentiality and anonymity.

## Results

30 critical care nurses and 20 physicians (adult intensivists, cardiologists, pediatric intensivists, neonatologists) from the ICUs, of all shifts, participated. 72% admitted to have practiced, at least once, dysthanasia and/or futile care, without a significant difference between in physicians (70%) and nurses (76.6%). 56% of respondents stated that the frequency with which they incur in any practice deemed by themselves as compatible with dysthanasia and/or futile care is 30% of their cases. The respondents obtained high or very high grades (from 86% to 98%) regarding their knowledge about: what a terminal illness is, what a terminal patient is, what dysthanasia is and what futile care is. 52% of respondents (physicians and nurses) reported not knowing the patients' rights. The right most mentioned by respondents (24%) was the right to die with dignity.

## Conclusions

72% of the ICUs' staff who was surveyed for this project affirmed to have practiced, at least once, dysthanasia and/or futile care. This was associated with their serious lack of knowledge about patients' rights (52%). This report should lead us to a deep reflection on the urgent need for addressing bioethical and humanization matters of the processes of education, training, supervision and practice of Intensive Medicine and Nursing.
